# Nanoscale Electromechanical and Conductive Properties of a Layered Two-Dimensional Hybrid Perovskite

**DOI:** 10.3390/ijms27177770

**Published:** 2026-08-30

**Authors:** Hee-Chang Jeon, Woohyuk Jang, Jiseon Yun, Sein Min, Joong Yeon Lim, Young-Seong Kim

**Affiliations:** Department of Mechanical, Robotics and Energy Engineering, Dongguk University, Jung-gu, Seoul 04620, Republic of Korea; hcjeon@dongguk.edu (H.-C.J.); jangwh21270@dgu.ac.kr (W.J.); ilyoung0430@dgu.ac.kr (J.Y.); molang0721@dgu.ac.kr (S.M.)

**Keywords:** two-dimensional hybrid perovskite, piezoresponse force microscopy, conductive atomic force microscopy, electromechanical response, resistive switching-like behavior, local charge transport

## Abstract

Two-dimensional (2D) organic–inorganic hybrid perovskites exhibit coupled ionic, electronic, and electromechanical responses that can strongly influence local charge transport. Here, solution-processed mixed-halide butylammonium lead perovskite crystals were mechanically exfoliated and investigated using X-ray diffraction, atomic force microscopy, piezoresponse force microscopy (PFM), and conductive atomic force microscopy (c-AFM). PFM measurements under −5, 0, and +5 V revealed clear bias-dependent changes in amplitude and phase, indicating an electric field-sensitive local electromechanical response. Local c-AFM measurements showed nonlinear bipolar hysteresis, with a pronounced increase in current near +7–8 V and a decrease near −7 to −6 V during the subsequent negative sweep. Because the crystals are mixed ionic–electronic conductors and the nanoscale tip–sample junction introduces substantial injection and contact barriers, the observed behavior is interpreted as resistive switching-like conductivity modulation, rather than definitive ferroelectric switching. The results are consistent with the combined contributions of charge injection, trap filling, possible ionic redistribution, and piezoelectricity-associated modulation of the local transport barrier. These findings provide nanoscale insight into electric field-dependent electromechanical and out-of-plane conductive behaviors in layered 2D hybrid perovskites.

## 1. Introduction

Two-dimensional (2D) organic–inorganic hybrid halide perovskites consist of molecularly layered structures in which semiconducting metal–halide inorganic layers are alternately stacked with bulky organic ammonium spacer cations [1,2,3]. This alternating organic–inorganic architecture gives rise to pronounced structural and dielectric anisotropy. The inorganic layers provide the primary pathways for charge transport, whereas the organic spacer layers regulate interlayer coupling, the dielectric environment, lattice distortion, and the energy barrier for out-of-plane charge injection [1,2]. In particular, the arrangement of organic cations and the structural distortion of the metal–halide octahedra can lower the local crystal symmetry, thereby inducing polarization and electromechanical responses that are sensitive to an external electric field [4,5,6,7].

Owing to these structural and electrical characteristics, 2D hybrid perovskites have attracted considerable attention as functional materials for resistive random-access memory (ReRAM) devices [2,4,8]. ReRAM is a nonvolatile memory technology in which the resistance state can be reversibly switched by an externally applied voltage. It offers several advantages, including a simple device architecture, high integration density, and low operating voltage [8,9]. Halide perovskites, in particular, have been extensively investigated as resistive switching materials because they can be processed from solution at low temperatures, exhibit coexisting electronic and ionic conduction, and allow their electrical properties to be tuned through compositional and structural engineering [8,9,10]. In early studies of organic–inorganic hybrid perovskite memories, electric field-driven vacancy or ion migration and the resulting modification of conductive pathways were proposed as the primary mechanisms responsible for resistance state switching [9,10].

More recently, the resistive switching characteristics of 2D and quasi-2D layered perovskites, in addition to those of 3D halide perovskites, have been actively investigated [2,3,4]. A quasi-2D (PEA)_2_Cs_3_Pb_4_I_13_-based device exhibited a lower high-resistance-state current, a higher ON/OFF ratio, and improved ambient stability compared with a 3D CsPbI_3_-based device [3]. Bipolar resistive switching has also been reported in the pure 2D Ruddlesden–Popper perovskites (TEA)_2_PbBr_4_ and (TEA)_2_PbI_4_, where the charge injection barrier and ion migration characteristics associated with the layered structure were interpreted as key factors governing the resistive switching behavior [2]. In addition, highly oriented (0 0 l) crystallographic alignment and multilevel resistive switching were reported in layered (C_6_H_5_CH_2_NH_3_)_2_CuBr_4_, suggesting that the arrangement of the organic spacers and inorganic layers can play an important role in controlling the conductive states [4]. These findings indicate that the 2D layered structure does not completely suppress charge and ion transport, but instead regulates their transport direction, interfacial barriers, and localized conductive pathways.

However, the electric field response of 2D lead–halide perovskites is difficult to explain solely in terms of the formation and rupture of conductive filaments. Lead–halide perovskites are mixed ionic–electronic conductors containing mobile halide ions, vacancies, and electronically active trap states; therefore, an external electric field can simultaneously induce the redistribution of ionic defects and injected charge carriers [10,11]. These processes can alter the local electrostatic potential, trap occupancy, and charge injection barriers at the organic–inorganic interfaces, consequently producing nonlinear and hysteretic current–voltage responses [10,11,12]. Accordingly, the local resistive switching characteristics of 2D hybrid perovskites should be understood as arising from the combined effects of charge injection, trapping and detrapping, ionic redistribution, and interfacial barrier modulation.

In addition, the orientation of organic cations, asymmetric displacement of Pb^2+^ ions, stereochemically active lone pairs, and distortion of the Pb–X octahedra can contribute to the local polarization and electromechanical response of 2D hybrid perovskites [5,6,7]. Recent studies have demonstrated that switchable polarization can be retained in 2D hybrid perovskites even at the thin-film or monolayer level, suggesting the possibility of electric field-driven control of polarization states within layered structures [13]. 

In a hybrid perovskite, piezoelectricity was also reported to modulate the current flow and resistance state of the conductive channel [14]. These findings suggest a possible coupling between piezoelectricity-related structural responses and local charge transport. Nevertheless, the relationship between electromechanical response and local conductive behavior in an individual 2D hybrid perovskite crystal remains insufficiently understood.

Piezoresponse force microscopy (PFM) enables the measurement of voltage-dependent amplitude and phase responses at the nanoscale and has, therefore, been widely used to investigate local electromechanical responses and polarization-related behavior [15]. In our previous studies, voltage-dependent changes in the piezoresponse phase and amplitude were observed in butylammonium-based 2D layered perovskites [5,6]. However, because metal–halide perovskites exhibit both ionic and electronic conduction, the measured PFM response may contain contributions not only from intrinsic polarization, but also from electrostatic interactions, charge injection, ionic redistribution, and electrochemical strain [16,17]. Therefore, rather than interpreting PFM results alone as definitive evidence of piezoelectricity-driven switching, they should be analyzed in conjunction with the corresponding electric field-dependent local conductive characteristics.

Conventional macroscopic electrical measurements average the responses arising from numerous grains, interfaces, defects, and electrode contacts, making it difficult to directly identify the local sites at which field-induced conductivity modulation occurs [15]. Conductive atomic force microscopy (c-AFM) enables the measurement of local current–voltage characteristics and current distributions through a nanoscale junction formed between a conductive tip and the sample, while simultaneously allowing the actual measurement location to be identified from the corresponding topographic image [18]. Therefore, the combined use of PFM and c-AFM provides a complementary approach for analyzing electric field-dependent electromechanical responses and local conductivity modulation in 2D perovskite crystals. Nevertheless, studies directly comparing local polarization-related responses with resistive switching characteristics in halide perovskites through correlated PFM and c-AFM measurements remain limited [19].

In this study, we extended previous investigations by examining the structure-dependent electromechanical and conductive properties of solution-processed 2D butylammonium lead–halide perovskites. First, the layered crystal structure and surface morphology were characterized using X-ray diffraction and atomic force microscopy. PFM was then employed to measure voltage-dependent changes in topography, amplitude, and phase in the piezoresponse phase and to evaluate the bias-dependent electromechanical response. In addition, c-AFM was used to investigate local nonlinear and hysteretic current–voltage behavior under bipolar voltage sweeps within individual 2D perovskite regions. By combining the PFM and c-AFM results, the observed local conductivity variations were interpreted as arising from the combined effects of organic–inorganic interfacial barriers, charge trapping, piezoelectricity-associated modulation, and possible ionic redistribution. This study provides nanoscale insights into the relationship between the molecularly layered structure of 2D hybrid perovskites and their electric field-dependent local charge transport behavior.

## 2. Results and Discussion

**Figure 1:**
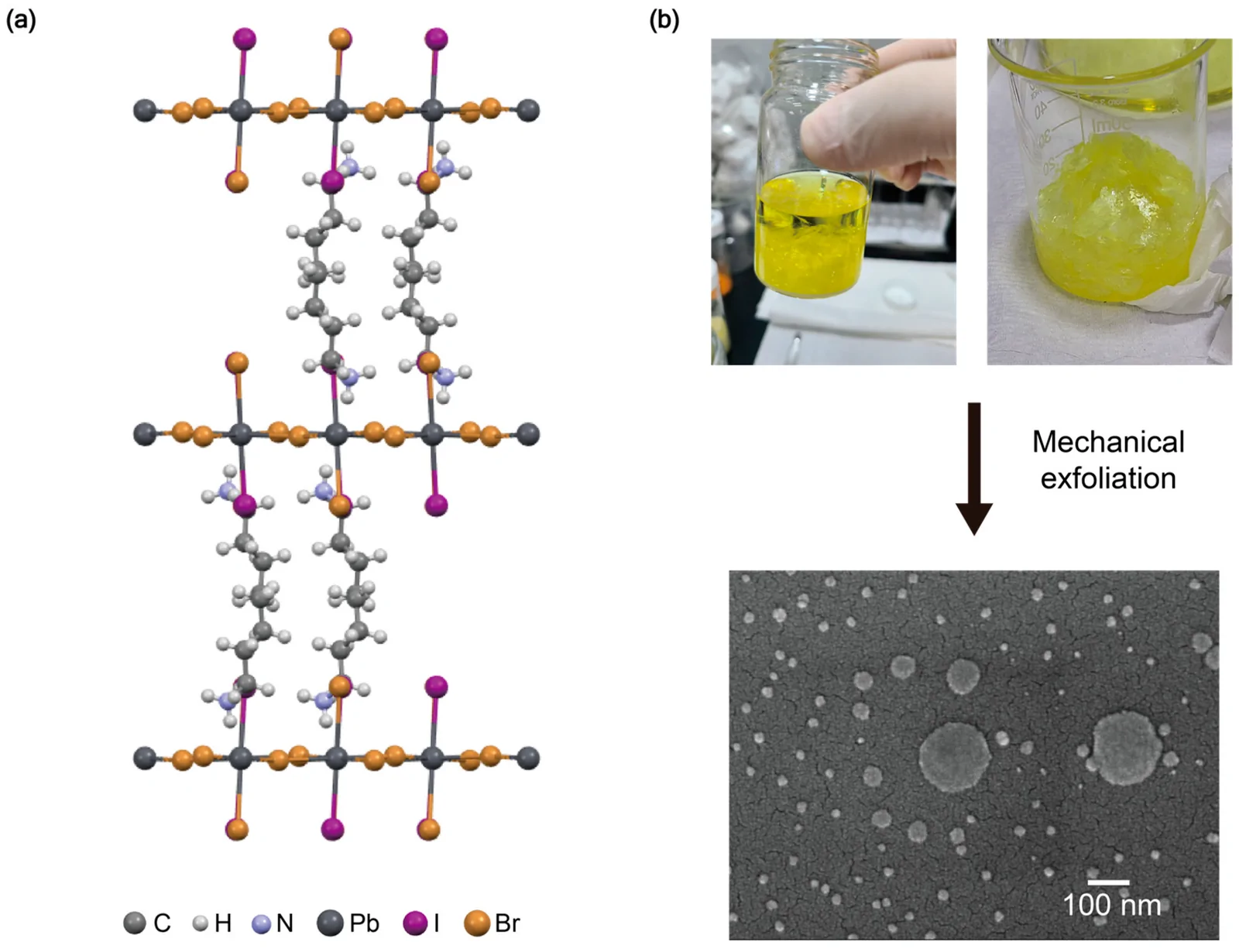
(**a**) Molecular structure determined by single-crystal X-ray diffraction (SC-XRD). (**b**) Photograph of the as-grown crystal and SEM image of the mechanically exfoliated crystal transferred onto a Si wafer.

Figure 1a illustrates the crystal formation process and the corresponding molecular structure of the synthesized material. The molecular model shows that bulky butylammonium cations form the organic spacer layers, while the metal–halide components constitute the inorganic framework. The halide composition was determined to be I_0.6_Br_3.4_, consistent with the precursor composition used for crystal growth.

The 2D organic–inorganic hybrid perovskite investigated in this study was fabricated through a solution-based process at a relatively low processing temperature. Owing to the presence of bulky butylammonium cations, the resulting crystal is expected to adopt an alternating organic–inorganic layered structure characteristic of 2D perovskites [1,2]. Such a molecularly layered architecture produces pronounced structural anisotropy and may influence out-of-plane charge injection, as well as the local electromechanical and piezoelectricity-related responses under an external electric field [1,5,6].

The layered crystal structure also results in relatively weak interactions between adjacent inorganic sheets separated by organic spacer layers [20]. Consequently, the synthesized crystals could be readily thinned through mechanical exfoliation, as shown in Figure 1b. This fabrication approach reduces the need for high-temperature thermal treatment and vacuum-based deposition processes. It therefore offers potential advantages for the fabrication of electronic devices on flexible and large-area substrates [21,22].

**Figure 2:**
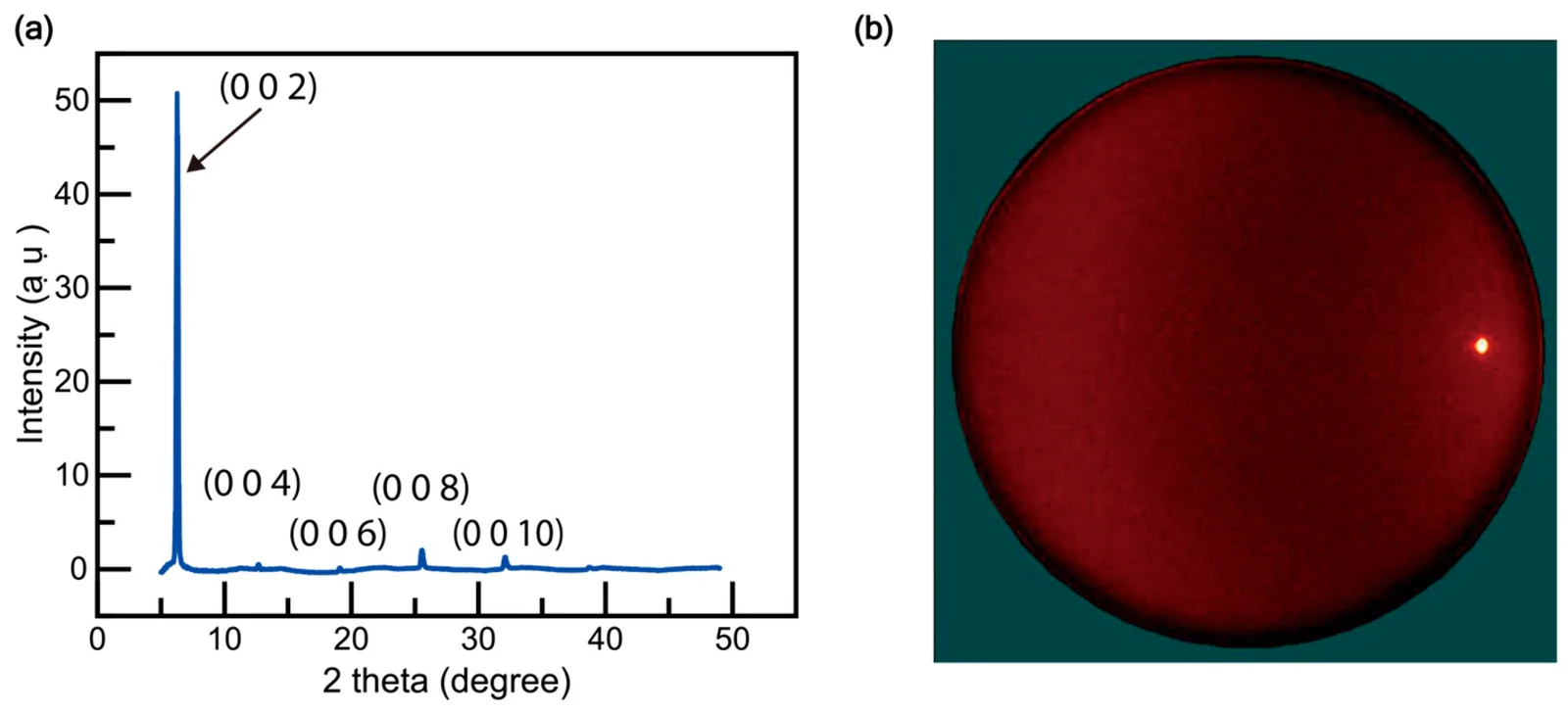
(**a**) Experimental X-ray diffraction (XRD) pattern and (**b**) optical microscopy image and high-resolution single-crystal X-ray diffraction (SC-XRD) results.

Figure 2a presents the X-ray diffraction (XRD) pattern of the synthesized 2D hybrid perovskite. The diffraction pattern exhibits a well-defined set of characteristic peaks without additional reflections attributable to secondary crystalline phases or impurities, indicating the formation of a phase-pure crystalline structure. The absence of multiple competing phases suggests that the solution-based synthesis produced structurally uniform and high-quality 2D hybrid perovskite crystals.

The formation of a well-defined layered crystalline phase is expected to promote a relatively uniform local electromechanical response. In particular, the structural orientation of the layered crystals may influence the direction and magnitude of the piezoresponse measured by PFM [23,24]. Therefore, the phase purity and crystallographic uniformity confirmed by XRD provide important structural bases for evaluating the piezoelectricity-related and bias-dependent electromechanical behaviors of the 2D hybrid perovskite.

**Figure 3:**
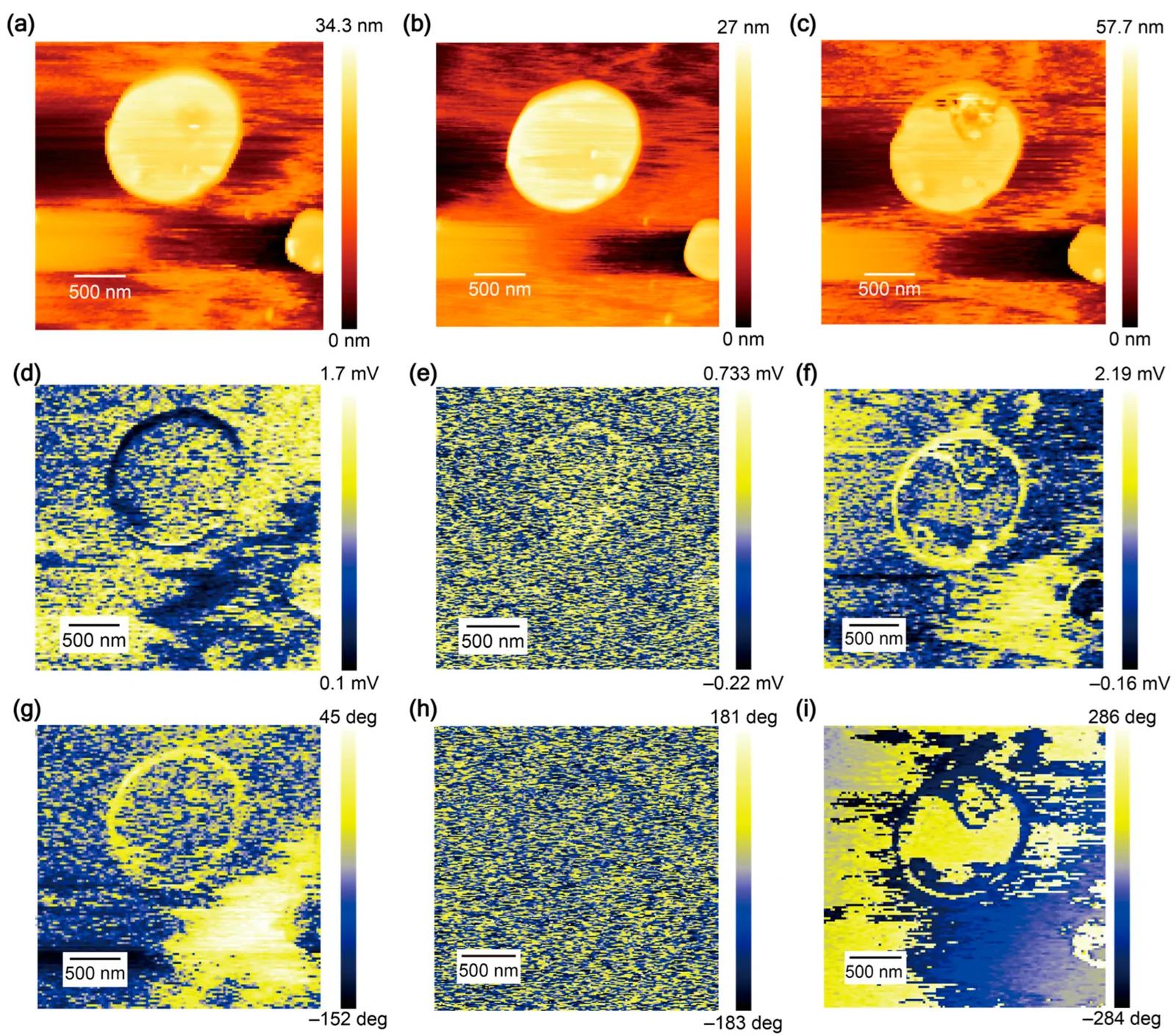
Bias-dependent PFM characterization of the mechanically exfoliated 2D hybrid perovskite crystal. (**a**–**c**) Topography, (**d**–**f**) PFM amplitude, and (**g**–**i**) PFM phase images measured at −5, 0, and +5 V, respectively.

Figure 3 presents the topography, PFM amplitude, and PFM phase images of the 2D perovskite crystal measured under applied DC biases of −5, 0, and +5 V. The mechanically exfoliated crystal exhibited a lateral dimension of approximately 2 μm and a thickness of approximately 20 nm, as determined from the AFM height profile measured before bias application (Figure S2). The overall shape and boundary of the crystal remained clearly identifiable under the different bias conditions, whereas pronounced variations were observed in the PFM amplitude and phase contrasts as the piezoresponse of the applied voltage was changed.

The PFM amplitude images in Figure 3d–f reveal bias-dependent variations in the magnitude and spatial distribution of the local electromechanical response. Similarly, the phase images in Figure 3g–i exhibit distinct contrast changes between the negative- and positive-bias conditions. The amplitude contrast represents changes in the magnitude of the local tip-induced electromechanical response, whereas the phase contrast reflects changes in the relative direction or phase of the response [15]. These results indicate that the local electromechanical and applied voltage-driven piezoelectric effects of the 2D perovskite crystal can be modulated by the magnitude and piezoresponse of the external electric field.

However, metal–halide perovskites are mixed ionic–electronic conductors, in which ionic and electronic transport coexist. Consequently, the measured PFM response may include contributions not only from piezoelectricity-related behavior, but also from electrostatic interactions, charge injection, ionic redistribution, and electrochemical strain [25]. The PFM results were, therefore, interpreted as evidence of an electric field-dependent local electromechanical and piezoelectricity-related response, rather than as standalone proof of intrinsic ferroelectric switching. This interpretation is consistent with our previous observations of voltage-dependent piezoresponse and phase modulation in butylammonium-based layered perovskites [5,6].

**Figure 4:**
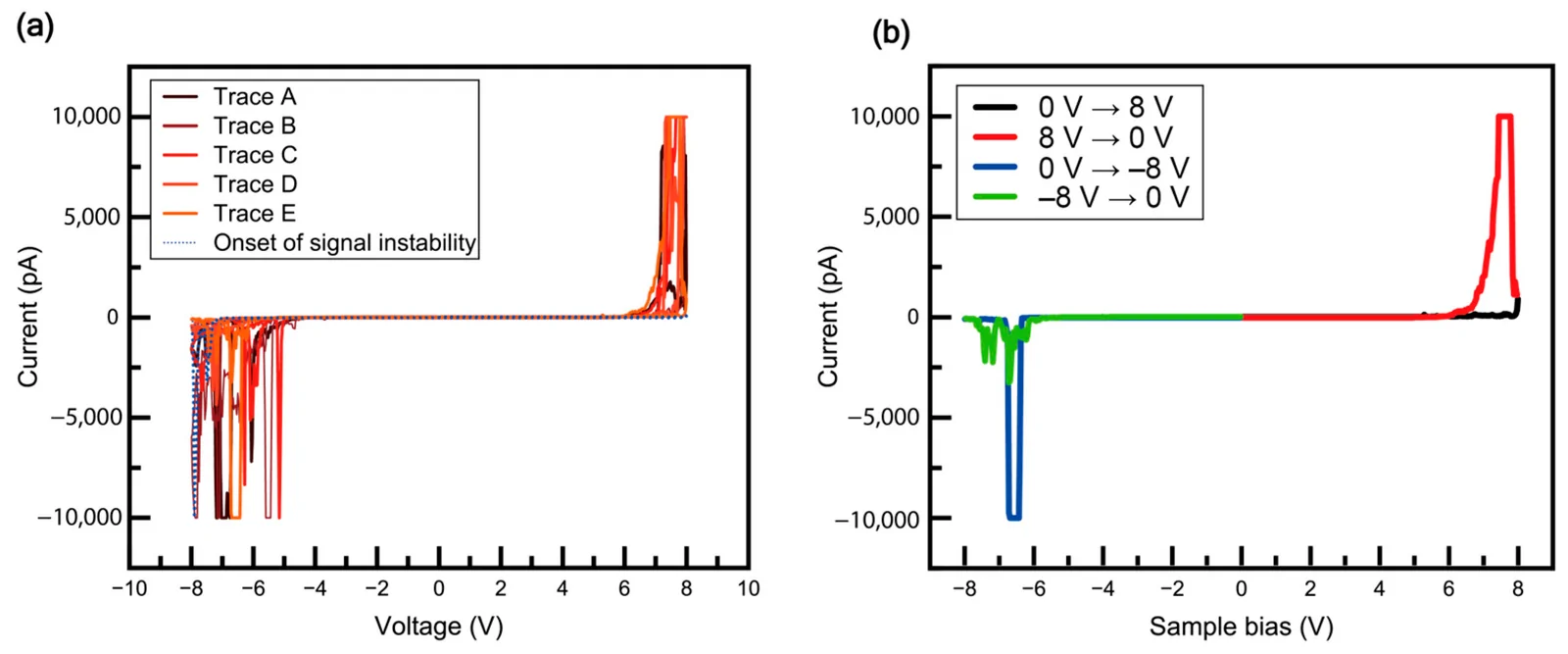
Local electrical characterization of the 2D hybrid perovskite using c-AFM. (**a**) Repeated local bipolar I–V traces (Trace A–E), showing the onset of signal instability during repeated measurements. (**b**) Local bipolar I–V characteristic of the 2D hybrid perovskite.

Figure 4 presents the local electrical characteristics of the 2D hybrid perovskite measured by c-AFM. During c-AFM characterization, the conductive tip acts as a nanoscale top electrode, enabling the out-of-plane charge transport characteristics of an individual 2D crystal to be measured between the tip and the underlying bottom electrode [15]. Unlike macroscopic I–V measurements, in which the electrical responses of multiple crystals, interfaces, defects, and electrode contacts are averaged, this nanoscale measurement enables conductivity variations within a specific 2D crystalline region to be directly investigated [15].

Additional repeated I–V measurements are presented in Figure 4a to evaluate the stability of the local electrical response. Although measurable hysteretic current responses were obtained during the initial measurements, the successive traces (Traces A–E) progressively changed and eventually exhibited pronounced signal instability. Comparison of the surface morphology before and after bias application also revealed local topographic modification (Figure S1). These observations are consistent with local electrical and structural degradation induced by the highly concentrated electric field and current density at the tip–sample junction [26]. Accordingly, repeated high-field biasing can modify the local electrical and structural states of the nanoscale crystal, preventing fully reproducible and reversible switching behavior from being maintained at the same measurement location.

The representative local I–V characteristic shown in Figure 4b was measured using the following bipolar voltage sweep sequence:

0 → +8 V→ 0 → −8 V → 0

Considering the maximum applied bias of 8 V and the approximate crystal thickness of 20 nm, determined before electrical bias application for c-AFM characterization (Figure S3), the nominal electric field estimated using E = V/t is approximately 0.4 V/nm. This value represents a nominal field estimate because the c-AFM bias is applied locally through a nanoscale conductive tip, resulting in a highly localized and non-uniform electric field distribution at the tip–sample junction, rather than the uniform field assumed in ideal parallel-plate geometry. Nevertheless, the relatively high applied field can induce substantial ionic redistribution, defect migration, charge trapping, and local structural modification in the hybrid halide perovskite [10,11].

During the initial positive-voltage sweep, the device remained in a low-current state, followed by a gradual increase in current with increasing bias. This progressive current increase may arise from carrier injection from the conductive tip and the subsequent filling of localized trap states within the 2D perovskite crystal [27]. At approximately +7–8 V, a pronounced nonlinear increase in current was observed, indicating a transition to a more conductive state.

After the maximum positive voltage was reached, the current followed a different path as the voltage was swept back toward 0 V. During the subsequent negative-voltage sweep, the relatively high-current state was maintained over a certain voltage range, followed by a decrease in current at approximately −7 to −6 V. Accordingly, the high-positive-voltage region can be regarded as an apparent SET region, whereas the high-negative-voltage region can be interpreted as an apparent RESET region.

Accordingly, although the initial bipolar I–V curve exhibits SET- and RESET-like transitions, these features should not be regarded as evidence of stable resistive switching. The high nominal electric field and the observed instability during repeated measurements indicate that field-induced ionic redistribution and local degradation may make substantial contributions to the hysteretic current response.

The observed behavior differs from the abrupt filamentary switching commonly reported in macroscale perovskite thin-film devices, where the formation and rupture of defect- or ion-mediated conductive filaments are frequently proposed as the dominant mechanisms [2,9,11]. In the present nanoscale 2D hybrid perovskite, the current initially increased progressively and exhibited a pronounced transition only at relatively high voltages. Considering the molecularly layered structure and nanoscale thickness of the crystal, the switching behavior may not be fully explained by a conventional metallic or defect-derived conductive filament model. Nevertheless, the relatively high switching voltage alone does not completely exclude field-induced ionic or defect redistribution because the local electric field distribution can also be influenced by the tip–sample contact geometry, organic–inorganic interfacial barriers, trap states, and contact resistance [28,29].

The PFM measurements revealed bias-dependent changes in electromechanical amplitude and phase, whereas the c-AFM measurements exhibited nonlinear and hysteretic conduction. These observations suggest that the local electrical response may involve coupling between field-sensitive structural changes and charge transport [13,14,15]. However, considering the high nominal electric field applied during the c-AFM measurements, field-induced ionic redistribution, defect migration, charge trapping, and local degradation are also expected to contribute substantially to the measured current response [10,11].

Therefore, the observed hysteresis is interpreted as arising from multiple field-dependent processes, rather than from a purely piezoelectric or electronic mechanism. Piezoelectricity-associated modulation of the local transport barrier may contribute to the response [13,14], while ionic redistribution, trapped-charge dynamics, and field-induced structural modification cannot be excluded and may become increasingly important under repeated high-bias operation [10,11].

**Figure 5:**
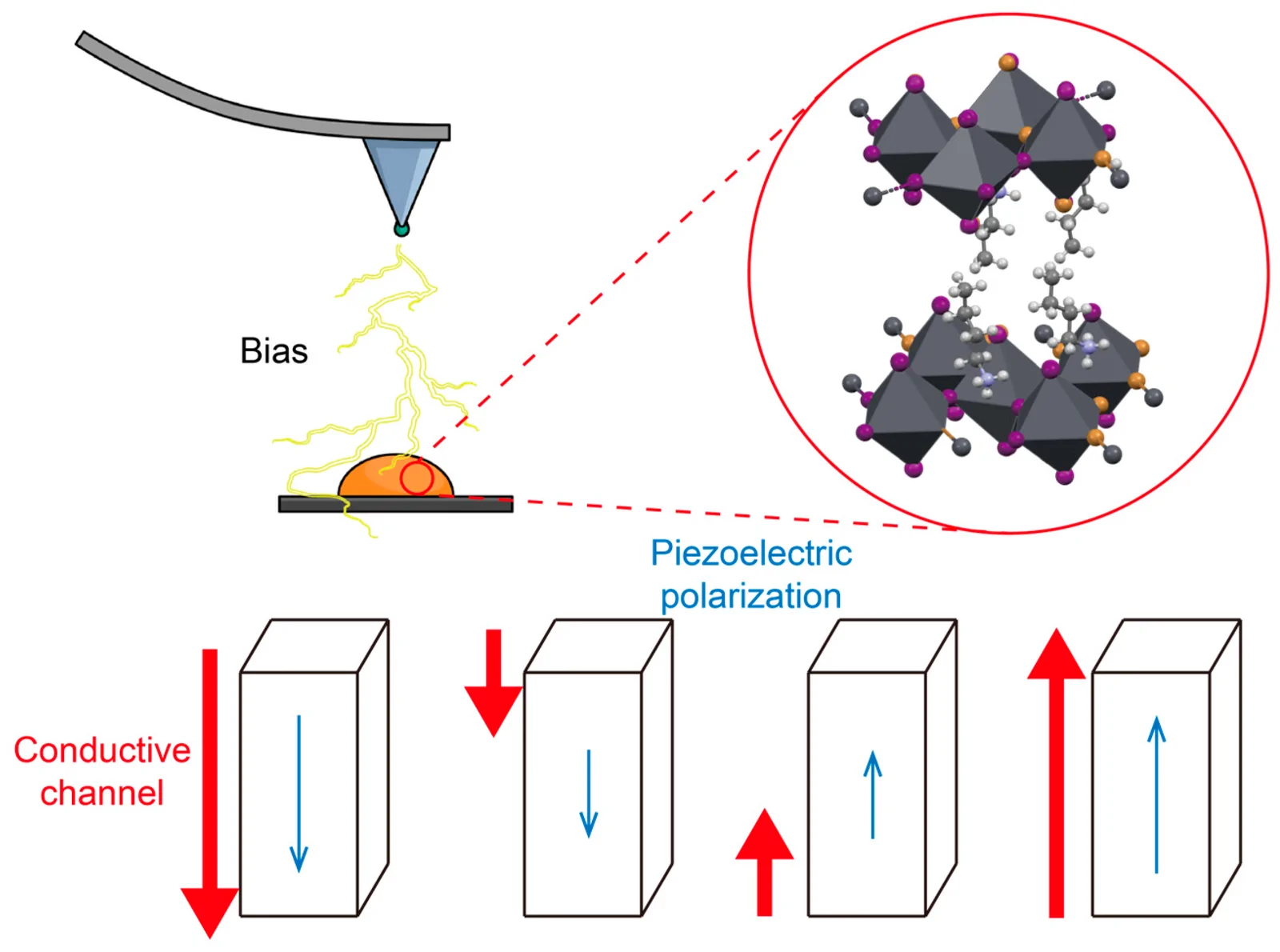
Schematic illustration of a possible piezoelectricity-associated contribution to the field-dependent local out-of-plane charge transport in the nanoscale 2D hybrid perovskite. Piezoelectricity-associated modulation of the local transport barrier is illustrated as one possible contribution, while ionic redistribution, charge trapping, and other high field-induced effects may also contribute to the observed conduction behavior.

A schematic representation of the possible coupling between the field-sensitive electromechanical response and local charge transport is presented in Figure 5. The schematic illustrates piezoelectricity-associated modulation of the local transport barrier as one possible contribution to the measured current response. Such field-sensitive structural and polarization responses have been reported in 2D hybrid perovskites [13,30]. However, under the relatively high electric fields employed in the present c-AFM measurements, ionic redistribution, charge trapping, and field-induced structural modification may also contribute substantially to the observed conduction behavior.

## 3. Materials and Methods

### 3.1. Materials

The mixed-halide butylammonium lead perovskite, (C_4_H_9_NH_3_)_2_PbI_0.6_Br_3.4_, was prepared using 1-butylamine (99%, C_4_H_9_NH_2_), hydriodic acid (47%, containing 1.5% hypophosphorous acid), lead(II) bromide (≥98%), anhydrous diethyl ether stabilized with butylated hydroxytoluene, ethanol (95%), γ-butyrolactone (GBL, 99%), *N*,*N*-dimethylformamide (DMF, ≥99.8%), dichloromethane (99.9%), and hexane (≥98%). Lead(II) bromide was purchased from ACROS Organics (Geel, Belgium). 1-Butylamine, hydriodic acid, DMF, and hexane were obtained from Alfa Aesar (Ward Hill, MA, USA). Diethyl ether, GBL, dichloromethane, and ethanol were supplied by TCI Chemicals (Tokyo, Japan), Daejung Chemicals (Siheung, Republic of Korea), Duksan Pure Chemicals (Ansan, Republic of Korea), and Samchun Pure Chemical Co., Ltd. (Pyeongtaek, Republic of Korea), respectively. All chemicals were used as received, without further purification.

### 3.2. Growth of (C_4_H_9_NH_3_)_2_PbI_0.6_Br_3.4_ Crystals

Butylammonium iodide (BAI, C_4_H_9_NH_3_I) was synthesized using a procedure adapted from our previously reported method, with the precursor composition subsequently adjusted for the present mixed-halide crystal. An appropriate volume of ethanol was first placed in a reaction vessel maintained in an ice bath. Hydriodic acid and 1-butylamine were then added at the required molar ratio, and the reaction mixture was stirred for 30 min. The solution was subsequently transferred to an oil bath and maintained at 70 °C for 2 h. Residual solvent was removed by heating the reaction mixture in a vacuum oven at 90 °C for 20 min. The resulting BAI powder was collected and washed several times with diethyl ether using vacuum filtration, followed by drying under vacuum at 70 °C for 12 h.

For crystal growth, DMF and GBL were mixed in equal volumes of 5 mL each. The synthesized BAI precursor was dissolved in the mixed solvent, after which PbBr_2_ was added according to the stoichiometric ratio required to obtain (C_4_H_9_NH_3_)_2_PbI_0.6_Br_3.4_. The solution was continuously stirred until a homogeneous and transparent yellow solution was obtained.

Crystal growth was carried out using a vapor-assisted antisolvent method. The precursor solution was placed inside a sealed container containing dichloromethane, without direct contact between the precursor solution and the liquid antisolvent. Slow diffusion of dichloromethane vapor into the precursor solution induced crystallization at room temperature. After 3 days, transparent yellow plate-like crystals were obtained. The crystals were collected by vacuum filtration, washed with hexane, and dried under ambient conditions.

### 3.3. Preparation of 2D Perovskite

The as-grown plate-like crystals were mechanically exfoliated to obtain thin 2D crystals. An individual bulk crystal was placed on Scotch tape and repeatedly cleaved using transparent adhesive tape. The exfoliation procedure was repeated eight times until little to no visible crystal residue remained on the upper tape. The resulting thin crystal flakes were then transferred onto either a Si wafer or a glass substrate by gently pressing the tape against the substrate and subsequently removing it.

### 3.4. Characterization

The crystal structures of the exfoliated samples were examined by X-ray diffraction (XRD). The samples were transferred onto glass substrates and analyzed at the Daegu Center of the Korea Basic Science Institute using a high-resolution X-ray diffractometer (D8 DISCOVER, Bruker AXS GmbH, Karlsruhe, Germany) with Cu Kα radiation. The X-ray optical configuration comprised a Montel mirror and a VANTEC-500 area detector, with a sample-to-detector distance of 301.1 mm.

The surface morphologies of the mechanically exfoliated crystals were examined using field-emission scanning electron microscopy (FE-SEM; JSM-7800F Prime, JEOL Ltd., Akishima, Tokyo, Japan). Atomic force microscopy measurements were performed at room temperature using a scanning probe microscope (NX10, Park Systems, Gwacheon, Republic of Korea). The surface topography and thickness of the exfoliated crystals were determined from AFM images and corresponding line profiles.

Piezoresponse force microscopy (PFM) measurements were conducted using a Multi75E-G cantilever to investigate the bias-dependent electromechanical amplitude and phase responses of the 2D perovskite crystals. PFM images were acquired over a scan area of 3 × 3 μm^2^ at a scan rate of 0.5 Hz. The topography, PFM amplitude, and PFM phase responses were measured under applied DC biases of −5, 0, and +5 V. Conductive atomic force microscopy (c-AFM) measurements were performed using a conductive CONTSCPt cantilever(NANOSENSORS, NanoWorld AG, Neuchâtel, Switzerland) to evaluate the local current–voltage characteristics of individual 2D perovskite crystals. Local bipolar I–V curves were acquired over a voltage range of −8 to +8 V using the sequence 0 → +8 V → 0 → −8 V → 0. The total sweep time for one bipolar I–V measurement was 1 s. The cantilever set point was 5.38 μN, with the Z-servo feedback maintained during measurement. Repeated I–V measurements were performed to evaluate the stability of the local electrical response. All measurements were conducted under ambient laboratory conditions, without active temperature or humidity control.

## 4. Conclusions

In this study, the electric field-dependent local electromechanical and conductive properties of solution-processed 2D butylammonium lead–halide perovskites were investigated using PFM and c-AFM. The PFM measurements revealed bias-dependent variations in amplitude and phase, indicating an electric field-sensitive electromechanical and piezoelectricity-related response. The c-AFM measurements showed bipolar hysteretic I–V behavior, characterized by a pronounced current increase at approximately +8 V and a current decrease at approximately −8 V during the subsequent negative sweep.

The observed resistive switching-like behavior is attributed to the combined effects of charge injection, trapping and detrapping, possible ionic redistribution, and piezoelectricity-associated modulation of the local transport barrier, rather than solely to a conventional conductive filament mechanism. These results provide nanoscale insight into the coexistence of field-sensitive electromechanical responses and out-of-plane conductivity modulation in 2D hybrid perovskites. Further studies using controlled current compliance and optimized electrode geometries are required to evaluate endurance and retention while minimizing tip-induced degradation.

## Data Availability

The data presented in this study are contained within the article.

## Supplementary Materials

The following supporting information can be downloaded at https://www.mdpi.com/article/10.3390/ijms27177770/s1.
