# Supplementary material for: Nanoscale Electromechanical and Conductive Properties of a Layered Two-Dimensional Hybrid Perovskite
Source: Int J Mol Sci. 2026 Aug 30;27(17):7770. doi: 10.3390/ijms27177770 (PMC13566253; doi:10.3390/ijms27177770)
Supplement: Supplementary file 1 [file ijms-27-07770-s001.zip › ijms-4510089-supplementary.pdf]

Supplementary Materials

Nanoscale Electromechanical and Conductive Properties of a Layered Two-Dimensional Hybrid Perovskite

Hee-Chang Jeon [1], Woohyuk Jang [1], Jiseon Yun [1], Sein Min [1], Joong Yeon Lim [1,*] and Young-Seong Kim [1,*]

[1] *Department of Mechanical, Robotics, and Energy Engineering, Dongguk University-Seoul, Jung-gu, Seoul 04620, Republic of Korea.*

*Corresponding author e-mail: jylim@dongguk.edu (J.Y.L.); kys4865@dgu.ac.kr (Y.-S.K.)

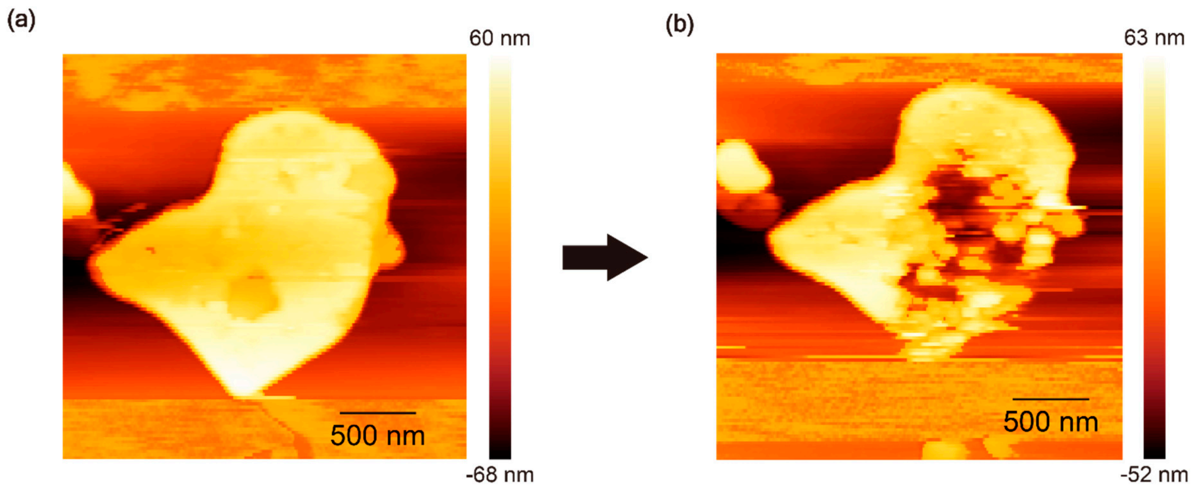

Figure S1. Surface morphology of the 2D hybrid perovskite sample before and after electrical bias application during c-AFM measurements. (a) Surface morphology before c-AFM measurement. (b) Surface morphology after c-AFM measurement.

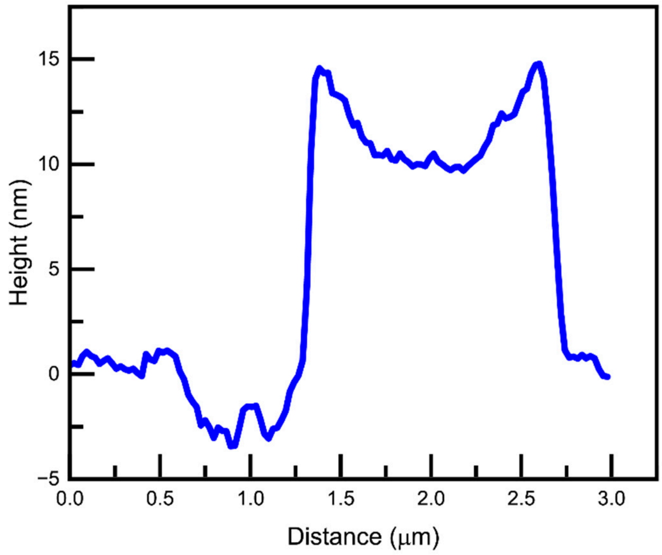

Figure S2. AFM height measurement of the pristine 2D hybrid perovskite sample before bias application for determination of the crystal thickness.

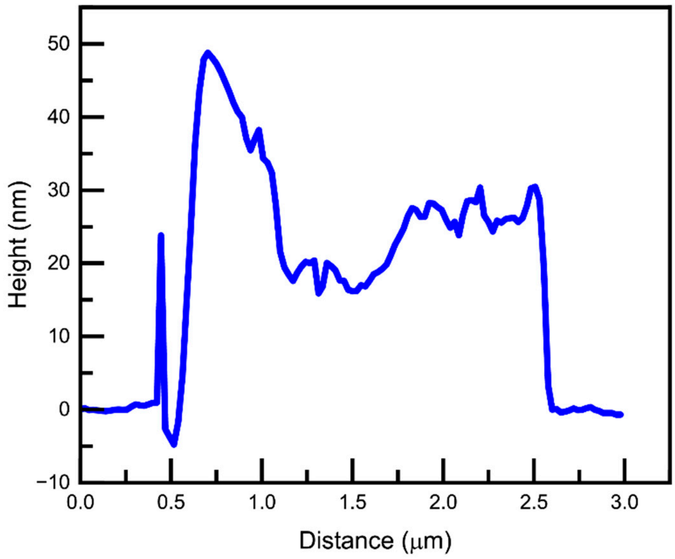

Figure S3. AFM height measurement of the 2D hybrid perovskite sample before electrical bias application for c-AFM characterization.
